# Gallium oxide nanowires for UV detection with enhanced growth and material properties

**DOI:** 10.1038/s41598-020-78326-x

**Published:** 2020-12-08

**Authors:** Badriyah Alhalaili, Ryan James Bunk, Howard Mao, Hilal Cansizoglu, Ruxandra Vidu, Jerry Woodall, M. Saif Islam

**Affiliations:** 1 Nanotechnology and Advanced Materials Program, Kuwait Institute for Scientific Research, Kuwait City, Kuwait; 2 Department of Electrical and Computer Engineering, University of California, Davis, Davis, USA; 3 Faculty of Materials Science and Engineering, University of Politehnica of Bucharest, Bucharest, Romania

**Keywords:** Electronic properties and materials, Nanoparticles, Nanowires, Electronic devices, Nanosensors

## Abstract

In the last decade, interest in the use of beta gallium oxide (β**-**Ga_2_O_3_) as a semiconductor for high power/high temperature devices and deep-UV sensors has grown. Ga_2_O_3_ has an enormous band gap of 4.8 eV, which makes it well suited for these applications. Compared to thin films, nanowires exhibit a higher surface-to-volume ratio, increasing their sensitivity for detection of chemical substances and light. In this work, we explore a simple and inexpensive method of growing high-density gallium oxide nanowires at high temperatures. Gallium oxide nanowire growth can be achieved by heating and oxidizing pure gallium at high temperatures (~ 1000 °C) in the presence of trace amounts of oxygen. This process can be optimized to large-scale production to grow high-quality, dense and long Ga_2_O_3_ nanowires. We show the results of morphological, structural, electrical and optical characterization of the β-Ga_2_O_3_ nanowires including the optical bandgap and photoconductance. The influence of density on these Ga_2_O_3_ nanowires and their properties will be examined in order to determine the optimum configuration for the detection of UV light.

## Introduction

Technology involving the development of ultra-wide bandgap semiconductors such as β-Ga_2_O_3_ has received considerable attention, its unique combination of chemical stability and wide band gap facilitates diverse UV applications in nanoscale electronics and optoelectronics^1^ such as solar UV monitoring, astronomy, communications, and detection of missiles. Recently, there has been significant interest in UV photodetectors (PDs) because of civil, military, environmental, and industrial market needs to improve UV instrumentation that operate in extremely harsh environments. Therefore, many studies have been proposed to fabricate UV photodetectors with specialized features to both survive in harsh environments and detect the UV region of the spectrum while remaining blind to visible wavelengths.

β-Ga_2_O_3_ is an ideal candidate for visible-blind UV-light sensors, particularly for power electronics, solar-blind UV detectors, and device applications in harsh environments^2,3^. In particular, nanowires have high surface area, small diameter, internal scattering, and high photoconductive gain, which can allow UV photodetectors based on them to achieve high responsivity. Furthermore, nanowires can minimize the side effects of lattice and thermal mismatch during the growth process, which simplifies the production of high performance devices^4^. Additionally, one of the benefits of using nanowires is the ability to enhance light absorption and confine light to increase photosensitivity^5^. For example, the presence of metal nanoparticles with size less than 10 nm at the surface of nanowires induces localized surface plasmons which can be excited and enhanced Rayleigh scattering^6^. Moreover, nanowire photodetectors offer the possibility to isolate optical absorption and carrier transport paths^7,8^.

In this work, the high sensitivity, simple and inexpensive fabrication process demonstrated by our β-Ga_2_O_3_ nanowire UV photodetector makes it promising for use in deep-ultraviolet detection applications. The β-Ga_2_O_3_ nanowires for UV photodetection were prepared by thermal oxidation at 1000 °C using an Ag catalyst. We demonstrate the structure and morphology of the nanowires in addition to performing optical and electrical characterization of the nanowire's sensing properties.

## Methods

First, quartz substrates were cleaned with acetone and isopropyl alcohol, rinsed with deionized water, and dried with a stream of nitrogen gas. The quartz substrates used in this experiment were 500 μm thick and 15 mm in diameter. A silver thin film 5 nm thick was deposited on the cleaned samples using a Lesker sputtering system. Sample not coated with Ag films were given the same cleaning treatment.

**Figure 1:**
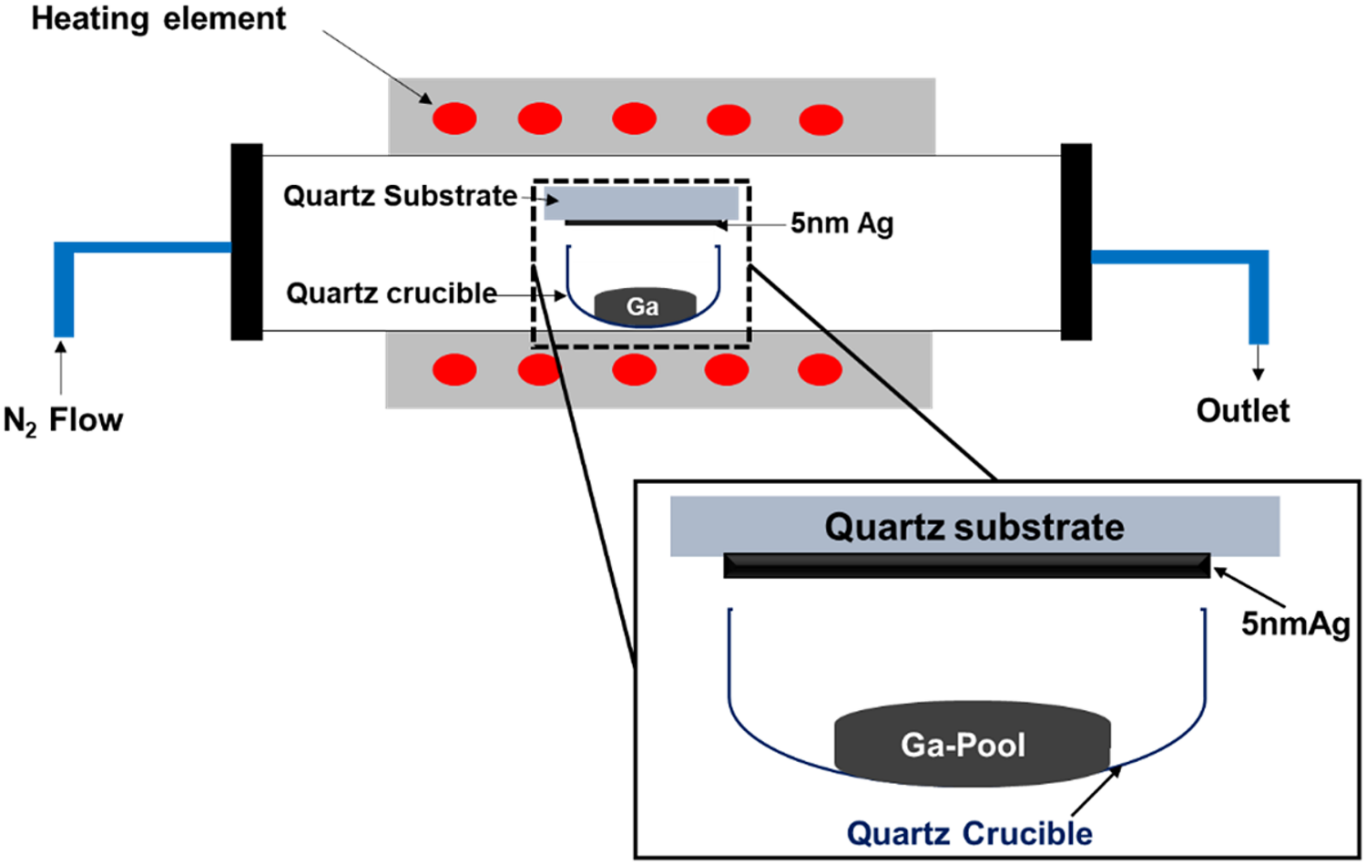
Schematic illustration of the UV photodetector fabrication technique.

Afterwards, the samples were re-cleaned with acetone and isopropyl alcohol, rinsed with deionized water, and then dried with an N_2_ gun to remove contaminants immediately prior to growth. Then, the samples were loaded into a quartz crucible which was placed into an OTF-1200X-50-SL horizontal alumina tube furnace made by MTI Corporation. The samples were exposed to temperatures of 800 °C and 1000 °C for 60 min. Heating occurred in a 20 sccm flow of nitrogen at atmosphere pressure. The background oxygen concertation was determined to be from 100 to 200 ppm using a downstream oxygen sensor. Samples were positioned above the gallium, such that the silver faced the gallium pool. The distance between the substrate and the gallium pool was about 10 mm. To minimize the potential for cross contamination, samples with silver and without silver were grown in different runs with dedicated crucibles. The quartz crucible was inserted inside the furnace, and the furnace was sealed and continuously flowed with nitrogen during growth. After the growth process, the electrical contacts were patterned on the top of the nanowires. A shadow mask was used to pattern 5 nm Cr and 50 nm Au on the surface of Ga_2_O_3_ Nanowires, deposited using a Lesker sputtering system. In addition, we examined the use of a 10 × 10 mm gold mesh as an external contact with 0.064 mm thickness. Figure 1 shows the growth apparatus of the Ga_2_O_3_ nanowires by Ag catalyst.

## Results and discussion

### Scanning electron microscopy (SEM)

**Figure 2:**
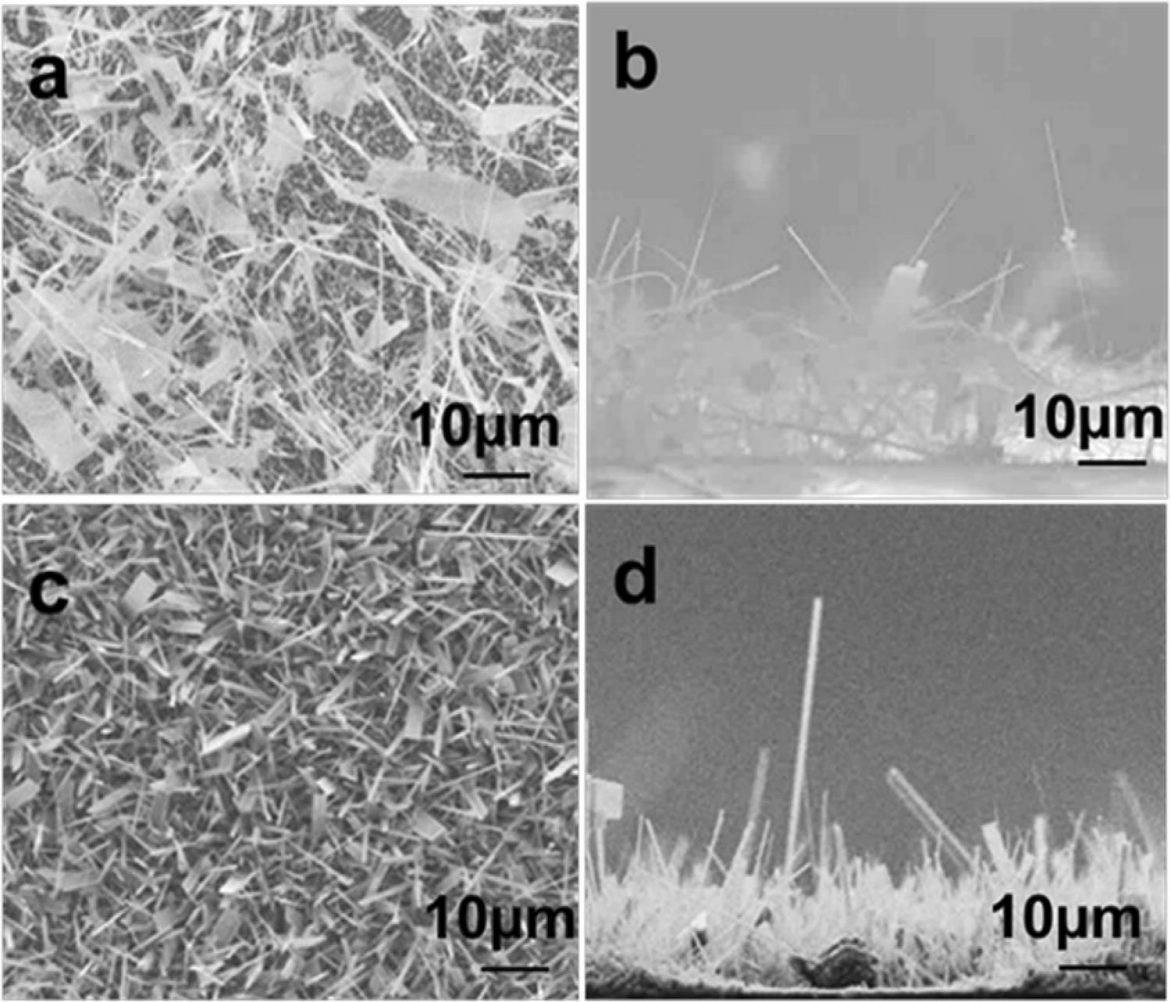
Ga_2_O_3_ nanowire grown at 1000 °C. (**a**,**b**) Top and side views of Ga_2_O_3_ on a quartz substrate, respectively. (**c**,**d**) Top and side views of Ga_2_O_3_ on quartz catalyzed by 5 nm Ag, respectively. Longer and denser NWs were achieved when Ag films are present on the sample before growth.

To explore the growth mechanism of Ga_2_O_3_ nanowires, Ga_2_O_3_ was grown with and without a 5 nm Ag thin film as a catalyst at 1000 °C. Interestingly, the morphology of the nanowires grown on bare quartz was different from that of the nanowires coated with 5 nm Ag. The Ag catalyst is found to enhance nanowire growth rate as well as increase the density of nucleation sites. SEM images (Fig. 2) showed that the density and size of the nanowires were much larger on Ag-coated samples after growth. Additionally, a homogeneous coating of Ga_2_O_3_ was observed under certain conditions and denser nanowires were grown at 1000 °C in the presence of 5 nm Ag compared to those grown without Ag. The Ag catalyst appears to play a role in reducing the diameters of the nanowires from 150–270 nm in the case of Ag-free growth to 120–160 nm when Ag was present. Even though denser, longer and thinner nanowires were observed by SEM imaging, no Ag NPs were seen at the tip or on the surface of Ga_2_O_3_ nanowires. Consequently, other characterization techniques were performed to investigate the morphology and elemental composition of these nanowires.

### XRD characterization

**Figure 3:**
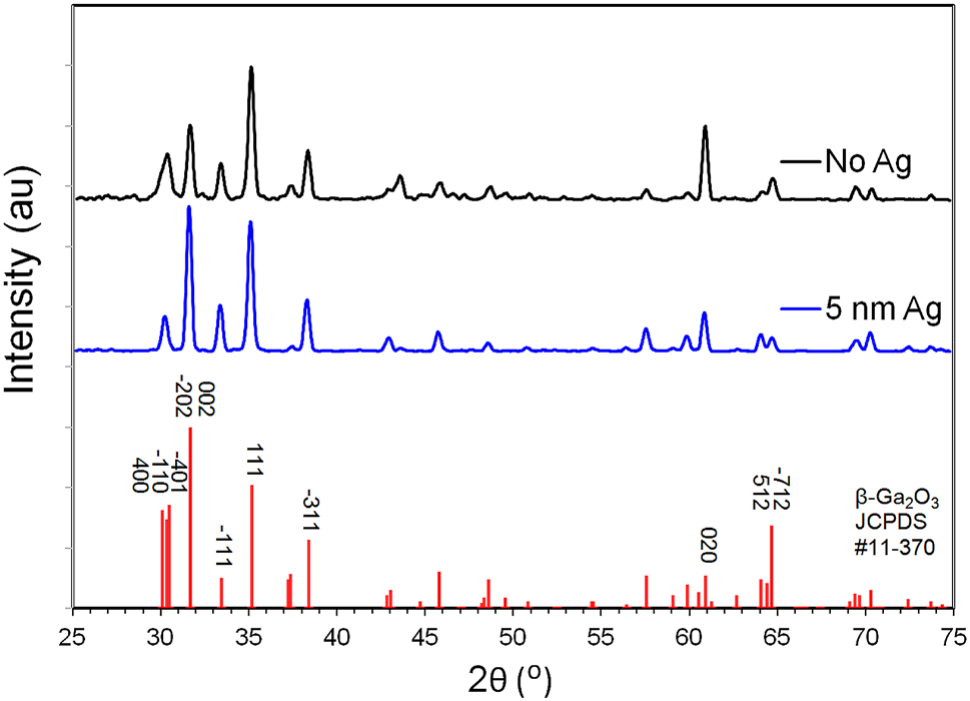
θ–2θ x-ray diffraction (XRD) pattern, indexed in comparison to JCPDS 11-370, for β-Ga_2_O_3_ grown at 1000 °C.

XRD was performed with a Panalytical XPert PRO Diffractometer (Malvern Panalytical, Netherlands). Figure 3 shows the XRD patterns of the Ga_2_O_3_ nanowire on quartz substrates oxidized at 1000 °C. The results are consistent with polycrystalline β-Ga_2_O_3_ results in the Joint Committee on Powder Diffraction Standards (JCPDS) card # 11-370. All major peaks of the β-Ga_2_O_3_ phase are seen to be present in the diffraction data, which strongly indicates the nanowires are β-Ga_2_O_3_. The apparent random orientation does not necessarily indicate the crystal growth direction is random, but more likely the nanowires are not self-aligned. However, it is noticeable that in the No Ag sample, the (020) peak is stronger and (002) peak weaker than random powder, and for the Ag sample it more closely follows a random pattern.

### Optical characterization

**Figure 4:**
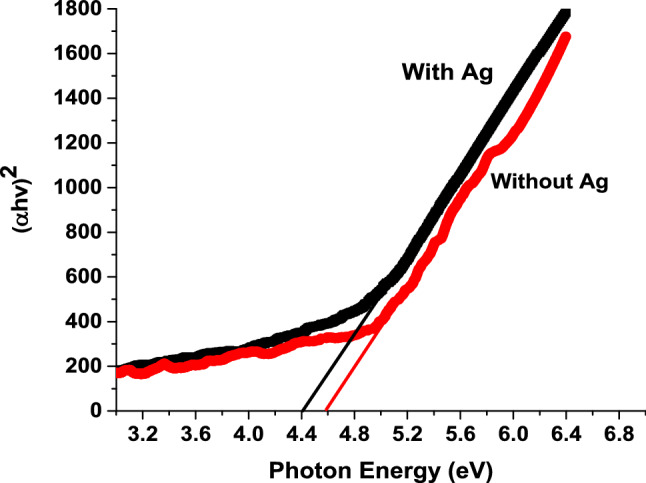
Plot of (αhv)^2^ versus photon energy.

A UV–VIS spectrophotometer (Ocean Optics HR4000CG-UV-NIR high resolution spectrometer) was used to measure the optical properties of the nanowires, such as transmission, reflection and absorption. To determine the bandgap of these nanowires, a plot of (αhv)^2^ versus photon energy was made. The bandgap was assessed by translating the transmittance curve and then correlating the bandgap (${E}_{g}$) with equation^9^

$${[\alpha hv]}^{2}=A(hv-{E}_{g})$$

where ${E}_{g}$ is the optical bandgap, A is a constant and $hv$ is the incident photon energy; α is the absorption coefficient. Figure 4. shows a plot of (*αhv*)^2^ versus photon energy. The bandgap of β-Ga_2_O_3_ nanowires with and without silver was found to be 4.6 eV and 4.4 eV, respectively. Table 1. shows previously reported results for the bandgap of β-Ga_2_O_3_ nanowires using different catalysts. Overall, the shift in the optical bandgap results which is about 0.2, which is in good agreement with the shift of the optical band gap (3.68 eV (Ag/Ga_2_O_3_) −3.85 eV (Ga_2_O_3_)) as reported previously^10^.

**Table 1:** Previously reported bandgaps of β-Ga_2_O_3_ nanowires.

Catalyst	Bandgap [eV]	Refs.
Ni	4.30	^11^
Au	4.7–4.8	^12^
	4.56	^13^
None	4.6	This work
5 nm Ag	4.4	

### Secondary Ion Mass Spectrometry (SIMS)

**Figure 5:**
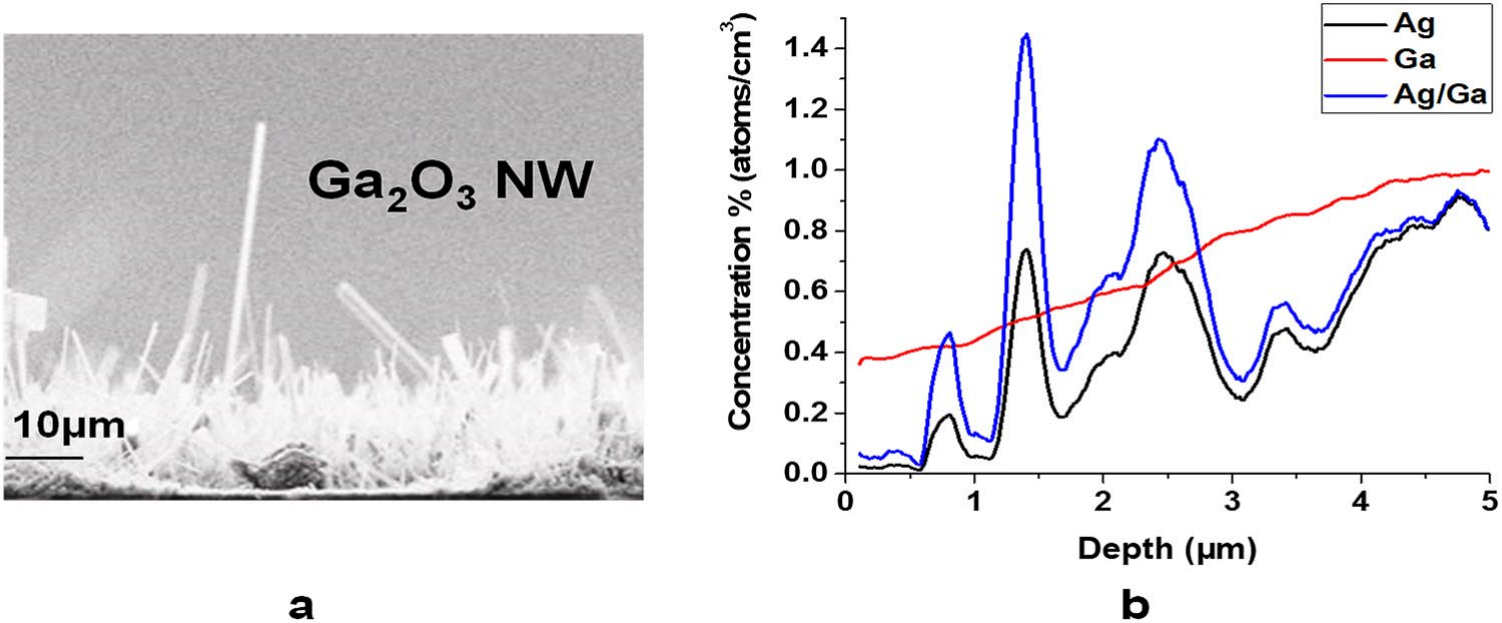
Ga_2_O_3_ NWs on quartz substrate by 5 nm Ag catalyst at 1000 °C. (**a**) SEM image. (**b**) SIMS profile. The SIMS was performed for nanowires from the tip to the base. As we go from the tip to the root of NWs, the concentration of Ag increases.

A SIMS profile analysis (Fig. 5) was performed to provide a better understanding of the composition of nanowires surface. The beam was incident normal to the surface of the sample. Due to the spot size limitation of the SIMS spectrometer, it was difficult to perform a single nanowire depth profile from tip to the base of nanowire, so the results are an aggregate of many randomly oriented and distributed nanowires. While the curve for Ga is relatively smooth, the curve of Ag has distinct peaks, demonstrating nonuniform spatial distribution of Ag within the nanowire forest. The ratio of Ag-to-Ga signal intensity versus depth has proportional increase towards the quartz surface.

### Scanning Transmission Electron Microscopy (STEM)

**Figure 6:**
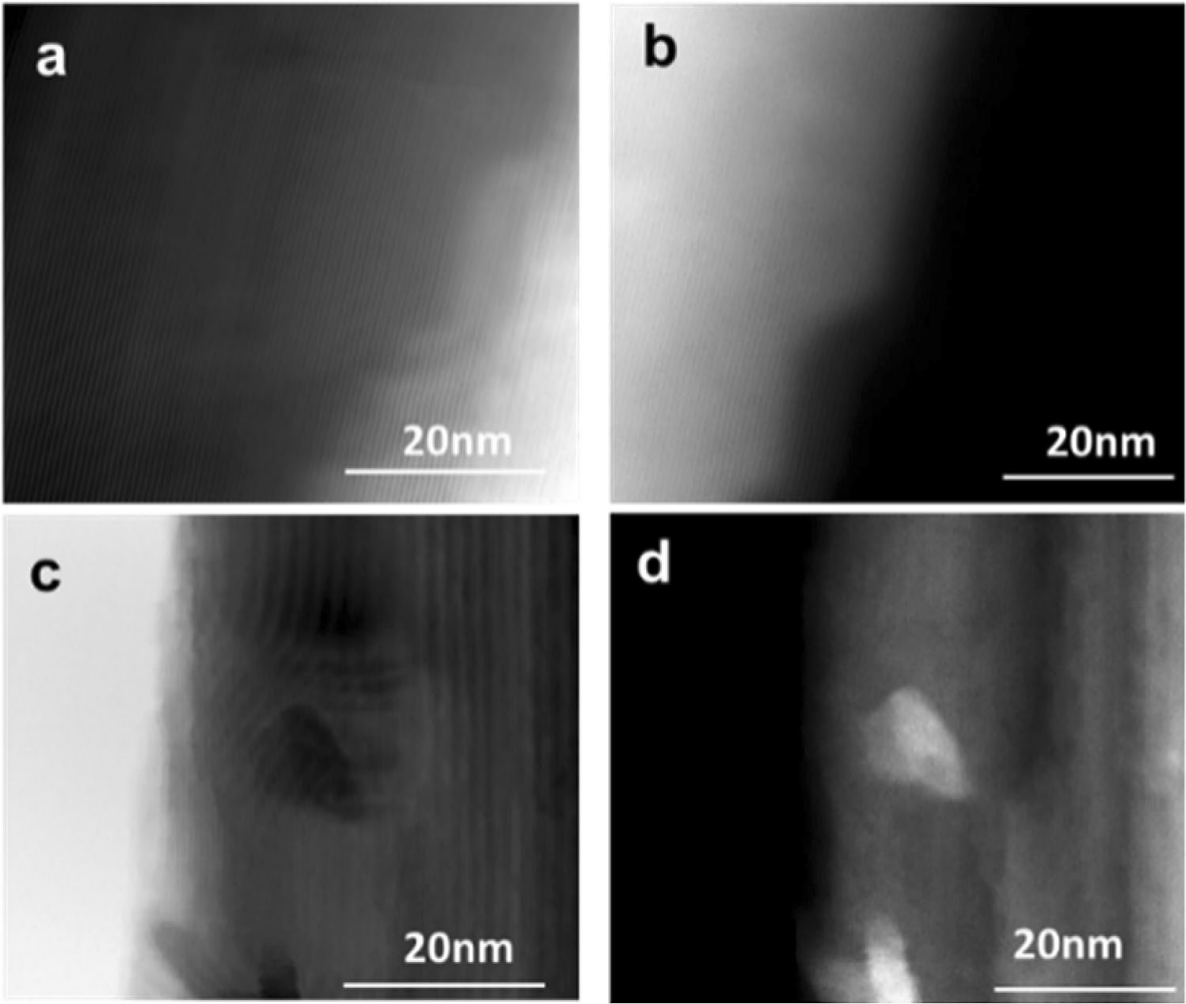
STEM images of Ga_2_O_3_ nanowire growth on quartz substrate at 1000 °C. (**a**) Bright field (BF) (**b**) high-angle annular dark-field (HAADF) STEM images of Ga_2_O_3_ nanowire without Ag catalyst. (**c**) Bright field (BF) (**d**) high-angle annular dark-field (HAADF) images of Ag NPs at the interface of Ga_2_O_3_ nanowire. The nanoparticle was bright due to z-contrast.

Figure 6 shows a STEM image of bright field (BF) and high-angle annular dark-field (HAADF) of a Ga_2_O_3_ nanowire grown at 1000 °C on quartz (Fig. 6a,b) and on quartz coated with 5 nm Ag (Fig. 6c,d). The β-Ga_2_O_3_ nanowire, which was not exposed to Ag catalyst, has no particles on its surface. However, the β-Ga_2_O_3_ nanowire shows several NPs with diameters between 5–10 nm that decorate the surface. Due to the transfer technique, it is not possible to determine the root or the tip of the nanowire. Han et al*.* has shown similar TEM characterization of Ag NPs on the surface of Ga_2_O_3_^10^. Bright field showed dark NPs due to diffraction contrast, since the nanoparticles are crystalline. Also, HAADF was performed to capture the z-contrast of Ag NPs. The NPs appear much brighter despite their small size, which suggests that their atomic mass and density are significantly larger than the surrounding β-Ga_2_O_3_, which points to Ag.

### High Resolution Transmission Electron Microscopy (HRTEM)

**Figure 7:**
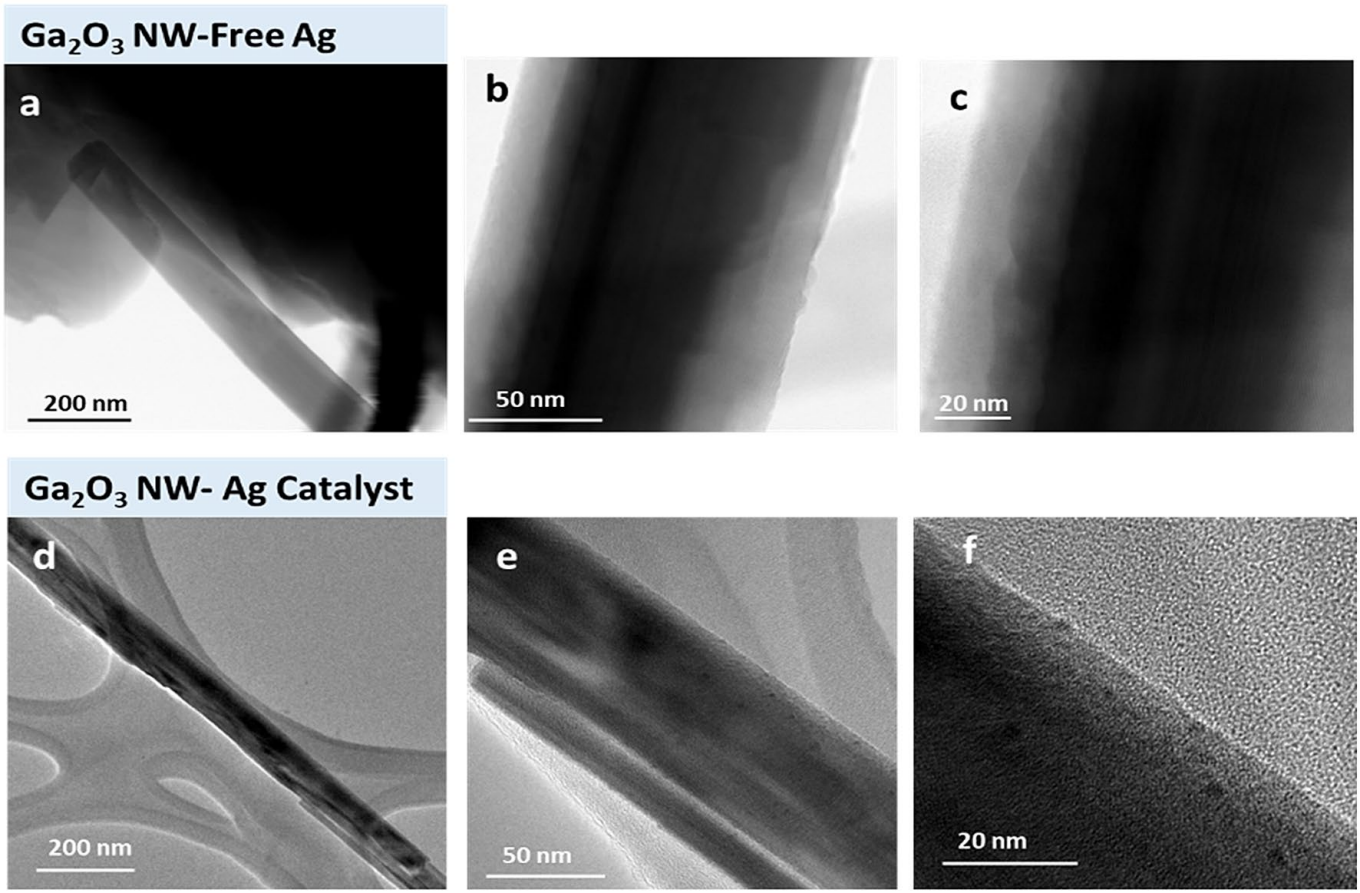
HRTEM images taken at different magnifications of single crystalline Ga_2_O_3_ NW grown on fused quartz substrate. The top row shows images of single Ga_2_O_3_ NW without the presence of Ag catalyst with scale bars at (**a**) 200 nm, (**b**) 50 nm and (**c**) 20 nm. Bottom row shows a Ga_2_O_3_ NW grown in the presence of Ag catalyst with different scale bars at (**c**) 200 nm, (**d**) 50 nm and (**f**) 20 nm. Few nanoparticles were observed on the surface of Ga_2_O_3_ NW as shown in (**d**) and (**f**).

To explore the presence of Ag on the surface of the grown Ga_2_O_3_ nanowires, a JEOL 2100F transmission electron microscope (TEM) was used to image Ga_2_O_3_ nanowires grown on a fused quartz substrate, without and with the presence of Ag as shown in Fig. 7. Ga_2_O_3_ nanowires without Ag catalyst show a smooth surface in HRTEM. However, Ga_2_O_3_ nanowire in the presence of Ag shows nanoparticles decorating the surface, with diameters between 2–5 nm.

**Figure 8:**
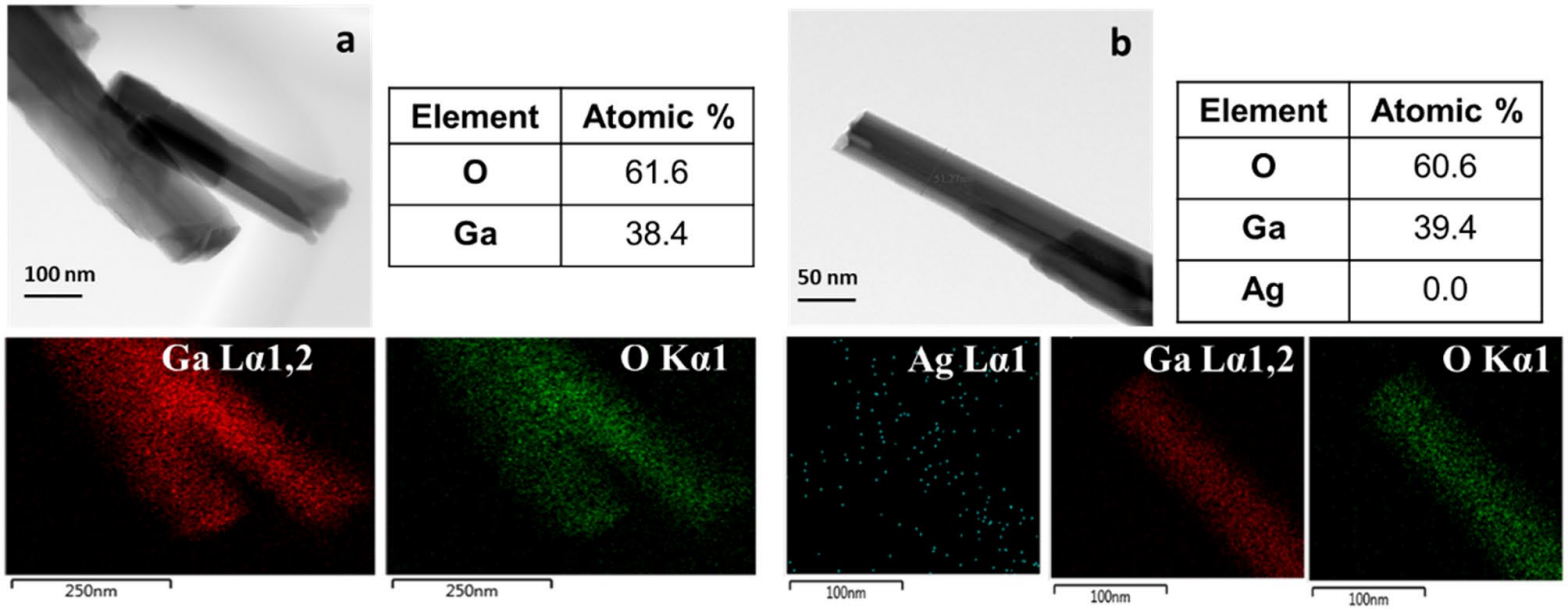
HRTEM image and the corresponding EDS mapping of Ga_2_O_3_ NW grown on quartz substrates (left) without the presence (right) in the presence of Ag catalyst.

The JEOL 2100F TEM was also equipped with an energy-dispersive spectroscopy (EDS) profile system, which was used to analyze the composition of Ga_2_O_3_ nanowires grown on quartz (Fig. 8). It was further used to search for silver, which was detected but not seen in the SEM observation. The TEM images correspond to the elemental mapping for Ga, O and Ag. Although no Ag nanoparticles were visibly detected on the surface of the nanowire, the particles may be too small to produce a signal detectable to the EDS detector in the TEM due to the large interaction volume required by EDS for electron capture and x-ray emission.

### Selective area diffraction pattern

**Figure 9:**
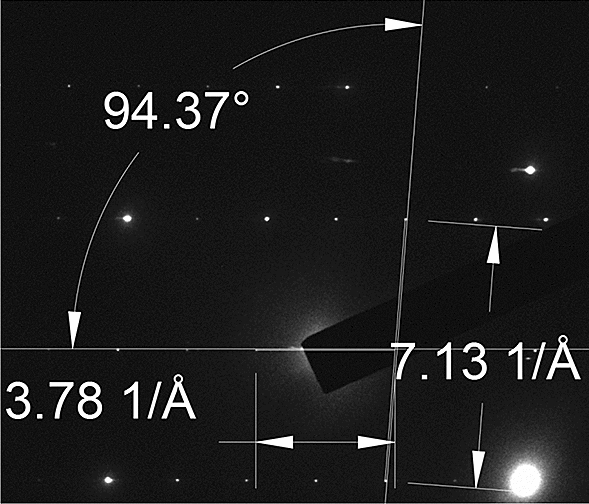
Measured angle and spacings for nanowire grown without Ag.

**Figure 10:**
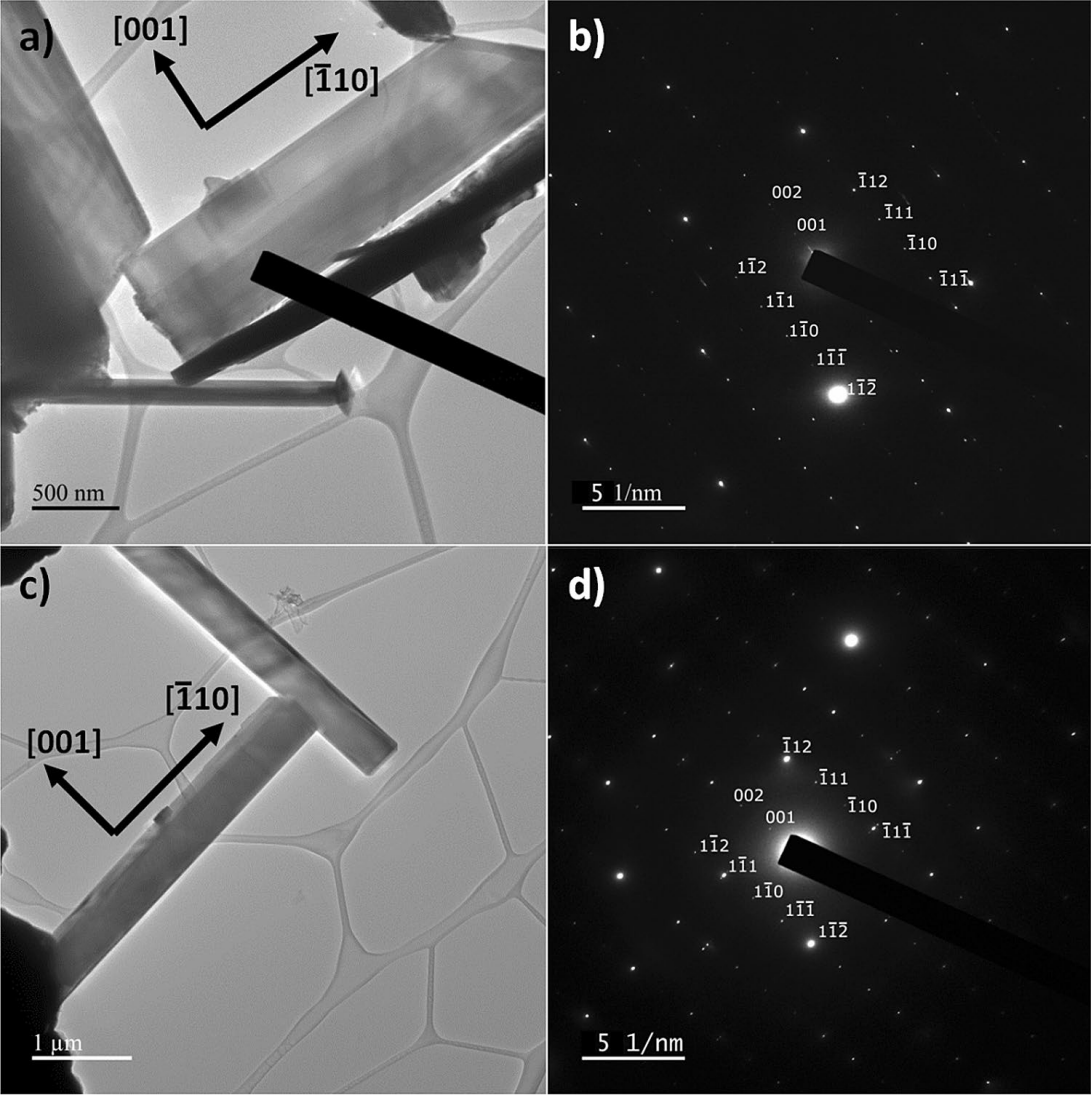
Indexed SAED pattern for (**a**,**b**) with Ag catalyst, (**c**,**d**) without Ag catalyst.

Selective area diffraction patterns were also taken to better understand the crystal structure of the nanowires obtained with or without Ag catalyst. The low-symmetry of the monoclinic crystal structure of β-Ga_2_O_3_ makes interpretation of diffraction results difficult in general, so the analysis of the patterns was guided using the SingleCrystal TEM diffraction simulator from CrystalMaker. To identify the zone axis, the spacings between points and the angles were measured, and compared to different zone axes, as shown in Fig. 9:

From the XRD results in Fig. 3, it is known in advance that the crystal structure of the nanowires is β-Ga_2_O_3_, which serves as a starting point for interpretation. It can be seen that the diffraction spots in Fig. 9 are distinct and sharp points, which strongly indicates the nanowires were single crystals. Using the lattice parameters of the β-Ga_2_O_3_ crystal, the pattern for various zone axes may be calculated. The result in Fig. 9 matches most similarly with the simulation for the [011] zone axis, with an angle of 94.05°, and a calculated [001] d/2 spacing of 3.5524 1/Å, and a calculated [–110] spacing of 6.7978 1/Å. Based on this, the image may be indexed using the simulation as a guide. The results of this are shown in Fig. 10.

In both cases the growth direction is found to be the [–110] direction based upon the measured diffraction spacing, with the orthogonal direction as [001]. This would indicate the difference in the [020] and [002] peaks shown in Fig. 3, which were not due to a difference in the orientation of the nanowires, but some other factor instead. Both samples growth with Ag and without Ag showed the same orientation, indicating that the growth acceleration due to Ag catalyst does not impact the orientation of the resulting nanowires. Additionally, there were no diffraction spots that could not be accounted for in this analysis, further indicating that not only were the nanowires single crystal, there are no twins or secondary Ga_2_O_3_ phases.

### Electrical characterization

#### Photocurrent and dark current measurements

**Figure 11:**
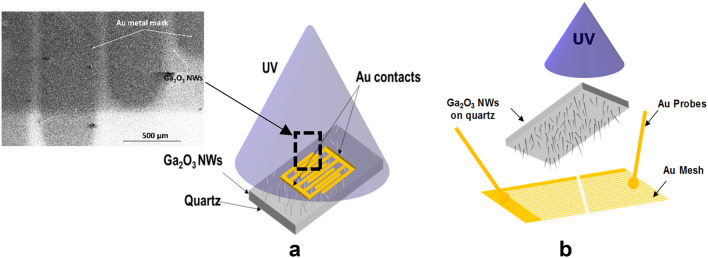
Schematic of Au/β-Ga_2_O_3_/Au metal–semiconductor-metal (MSM) photoconductor on quartz. (**a**) Sputtered gold contacts. The distance between the circles probes is 3 mm. Top-left SEM image of Au contacts at the surface of Ga_2_O_3_ NWs. (**b**) Au mesh gold contacts.

A Au/β–Ga_2_O_3_/Au metal–semiconductor–metal (MSM) photoconductor (Fig. 11a) was fabricated to investigate the electronic conductivity of the grown β-Ga_2_O_3_. The surface of Ga_2_O_3_ NWs was attached to a shadow mask to deposit Au/Cr contacts by using a Lesker sputtering system. For the electrical measurements, a custom probe station attached to a Keithley 2400 SMU was used and UV illumination was from a broadband Dymax Bluewave75 UV lamp (280–450 nm) (Dymax Corporation, USA). In addition, we performed other selective wavelengths from Newport monochromator with XeHg lamps.

**Figure 12:**
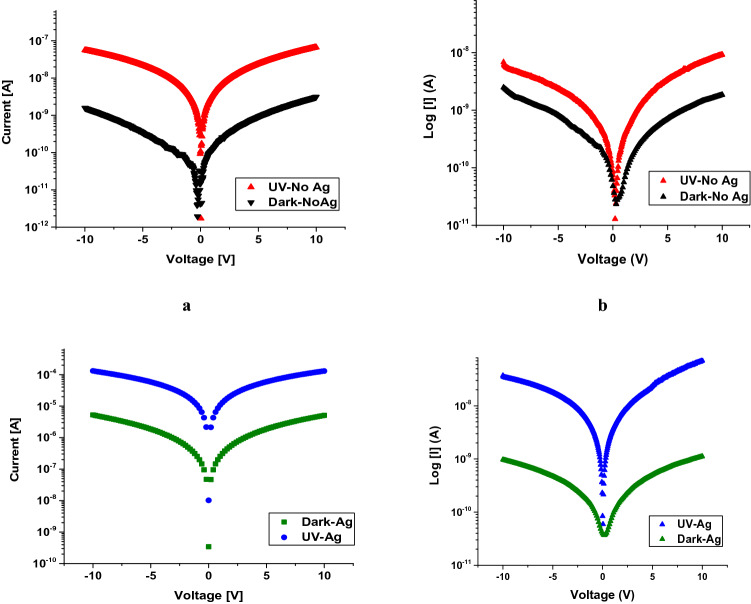
Semi-logarithmic plots of current density for Au/β-Ga_2_O_3_/Au MSM without and with Ag catalyst versus applied voltage characteristics at 10 V without and with UV illumination. (**a**,**c**) with sputtered Au metal contacts (**b**,**d**) with mesh Au metal contacts.

The current–voltage (*I–V*) characteristics were measured in dark conditions and under UV illumination for a MSM structure with and without an Ag catalyst at 10 V (Fig. 12). The MSM back-to-back Schottky diodes under UV illumination with a light intensity of 15 W/cm^2^ showed results comparable to the dark current. Due to the surface plasmonic effect of Ag NPs at the surface of Ga_2_O_3_ NWs, the absorption of photon is highly enhanced^14^, leading to more photoexcited carriers transport and collection at the contacts (Fig. 12c,d). Additionally, the results showed that Ga_2_O_3_ on quartz with a 5 nm Ag catalyst improves the photocurrent response to the broad spectrum UV source. The results reveal that the steady photocurrent of Ag/β–Ga_2_O_3_ (Fig. 12c) was three order of magnitude higher than that of the photocurrent of Ag-free/β-Ga_2_O_3_ (Fig. 12a). Hence, silver as a catalyst plays a critical role in improving the electrical properties of the β-Ga_2_O_3_ nanowires. The photodetection mechanism of the β-Ga_2_O_3_ nanowire is credited to different aspects that mainly include the incident light absorption, carrier photogeneration and carrier transport and surface oxygen adsorption and desorption process^15^.

The metal contacts deposition method has a large impact on the dark current and photocurrent. Even though Ag/Ga_2_O_3_ was sputtered with gold contacts shows high current response under illumination, it showed a large increase in the dark current as well (Fig. 12a,c). The major disadvantage of sputtering gold contacts is the induction of a damage that effects Fermi level pinning and hence the electrical measurements. This is due to the interfacial disorder of oxygen or Ag atoms vacancies^16^. Therefore, Au mesh contacts were used to avoid the consequence of sputtering technique as shown Fig. 11b. With using this technique, the results have been improved (Fig. 12b,d). The presence of Ag NPs at the surface was found to increase both photocurrent and dark current for sputtered contacts, however for mesh contacts it produced a substantial decrease in dark current. This could be due to an interaction between the Ag and deposited Au which produced a large leakage, but would not occur for mechanically applied mesh contacts.

The presence of Ag NPs at the surface plays a critical role in reducing the dark current of Ga_2_O_3_ on quartz compared to the one without Ag catalyst when mesh contacts are used, as shown in the Fig. 12. The results indicated that a covering of Ag nanoparticles on the Ga_2_O_3_ surface of devices can both decrease the dark current, and increase the photocurrent, both of which are beneficial. In addition, larger photocurrent can be observed for Ga_2_O_3_ on quartz decorated with Ag NPs. For dark current, a Schottky barrier formed at the interface between Ag and Ga_2_O_3_ due to the difference of the work function, where Ag NPs and Ga_2_O_3_ have the equal Fermi energy level. However, the positively charged Ag NPs depleted the carriers near the nanowires surface. This may deplete carriers in the nanowires in the absence of light, leading to higher dark resistivity in the material and lower dark current.

**Figure 13:**
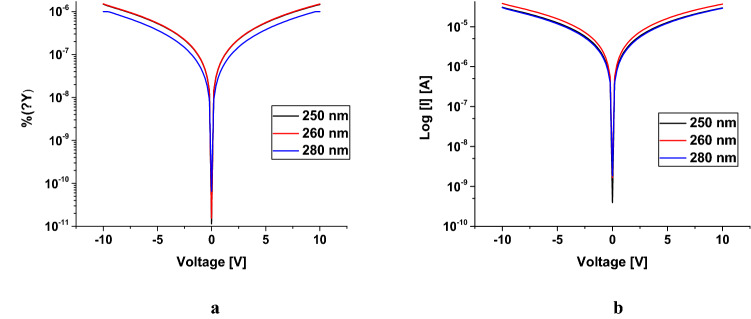
(**a**) semi-logarithmic plot of current–voltage for Au/β-Ga_2_O_3_/Au MSM with UV illumination at 10Vat different wavelengths such as 250 nm, 260 nm and 280 nm. (**a**) without catalyst (**b**) with Ag catalyst.

The I-V measurement of the photocurrent for the photodetector was measured at different selected wavelengths such as 250, 260 and 280 nm with 1mW LED lamps to evaluate the device performance for the detection of deep UV light with high input power (Fig. 13a). interestingly, the photodetection response with Ag catalysts was improved by almost two order of magnitude. The ratio of photocurrent to dark current was measured for the Ga_2_O_3_ on quartz without and with 5 nm Ag catalyst (Fig. 13b) at 10 V for Ga_2_O_3_ with Ag catalyst were almost 38.3 compared to the Ag-free Ga_2_O_3_ which was 4.55. Table 2 shows the performance of the developed β-Ga_2_O_3_ device compared to others.

**Table 2:** Summary of β-Ga_2_O_3_ device performance of present device and other previously reported UV PDs.

Device structure	MSM		NW network	MSM
	No Ag	5 nm Ag		
Fabrication method	Oxidation/vaporization		CVD	MOCVD
Electrode	Cr/Au		Au	Au
Light of detection (nm)	190–400		290–340	255–260
I_Photo_/I_Dark_	4.55@10 V	38.3@10 V	50@10 V	4.67
Year	2019		2016	2015
Reference	This work		^17^	^18^

**Figure 14:**
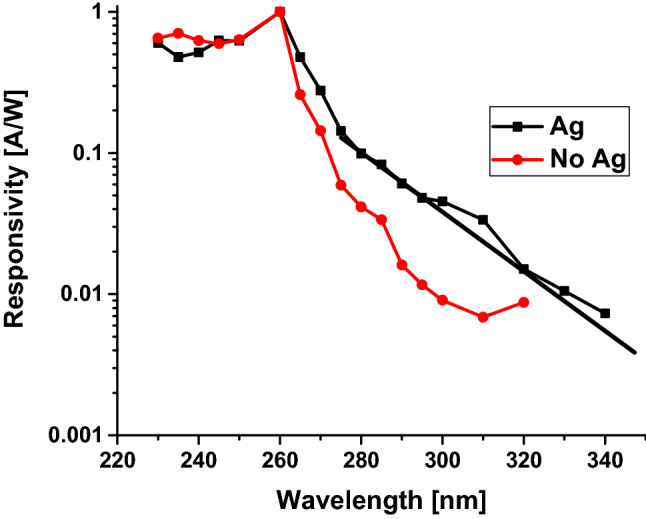
Responsivity vs different wavelengths for Ga_2_O_3_ without and with the presence of Ag catalyst.

Demonstration of UV photodetector responsivity based on a Ga_2_O_3_ nanowire without and with Ag catalyst was examined (Fig. 14). The responsivity of Ga_2_O_3_ on quartz with Ag catalyst was higher than the one without catalyst approximately by 1.5 order of magnitude. In addition, responsivity curve measurements were taken using a XeHg lamp coupled to a monochromator to obtain a responsivity curve, as well as the responsivity at solar wavelengths. This data is shown in Fig. 14, which shows a larger response over a broad range of wavelengths. The solar-range response (> 300 nm) is significant in both cases, however the relative responsivity in the Ag sample is about 3–5 times larger below the peak at 260 nm. To qualify a device's solar blindness, the solar-UV rejection ratio is typically used^19^, which in this case we define as the response at 320 nm, which is near the edge of the solar band, versus the response at 260 nm. For the Ag sample, the solar-UV rejection ratio is about 50, for the sample without Ag the rejection ratio is closer to 100.

When the incident UV light on the surface of β-Ga_2_O_3_ nanowire in the presence of Ag NPs is below 320 nm, the impact of interband transitions on Ag NPs occurs^20–22^. Hence, highly energetic hot electrons of Ag NPs will be excited from the 4d and 5sp bands. This property could enhance the performance of Ga_2_O_3_ in ultra-violet detection due to the presence of Ag NPs, which would lead to the observed shallower tail in the responsivity curve.

#### Transient photocurrent

**Figure 15:**
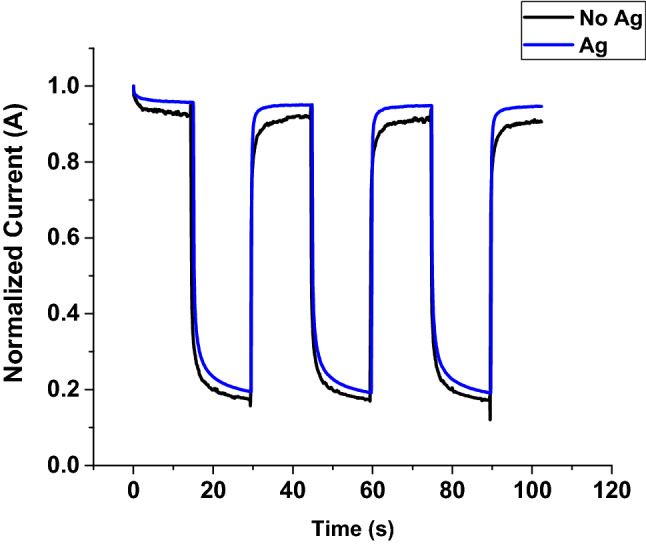
Transient response of the UV photodetector fabricated based on Au/β-Ga_2_O_3_/Au MSM at 5 V without and with Ag catalyst.

The transient response of the photodetector was measured by opening and closing a shutter on a broad spectrum UV light source (Fig. 15). Ga_2_O_3_ on quartz with an Ag catalyst showed a fast-transient response due to enhanced carrier transport. When the UV illumination was turned on, there was a large increase in the photocurrent. Due to the surface plasmonic effect of Ag NPs at the surface of Ga_2_O_3_ NWs, the absorption of photon is highly enhanced^14^, leading to more photoexcited carriers transport and collection at the contacts. On the other hand, when the UV light is turned off, the free electrons will recombine with holes very rapidly.

In Ga_2_O_3_ without an Ag catalyst, by turning the light on and off, a relatively slow response occurred in the device turning on. This slow response was attributed to the traps and surface states of oxygen generated at the surface of gallium oxide^23^. A longer tail in the photocurrent occurred when the light was turned off due to the reduction of charge carrier recombination as a result of captured hole-trap states. Hence, a longer recovery time is required because of the diffusion of oxygen molecules. The average rise time (from 10 to 90% of maximum photocurrent) and fall time (from 90 to 10% of maximum photocurrent) are 1.12 s and 2.0 s for Ag-free Ga_2_O_3_ and 0.33 s and 2.2 s for Ga_2_O_3_ in the presence of Ag. Hence, Ag NPs presence enhances faster rise time, but similar fall time. In Ga_2_O_3_ with an Ag catalyst, silver nanoparticles enhance the adsorption of light in the sample, and thus and desorption of oxygen molecules. When Ag NPs were excited by the UV light, each nanoparticle will generate a light-induced dipole^24^. Hence, the dipole-diploe interaction of Ag nanoparticles influences each nanoparticle and those nearby it.

### Silver (Ag) nanoparticles (NPs) catalyzes the growth of Ga_2_O_3_ nanowires

**Figure 16:**
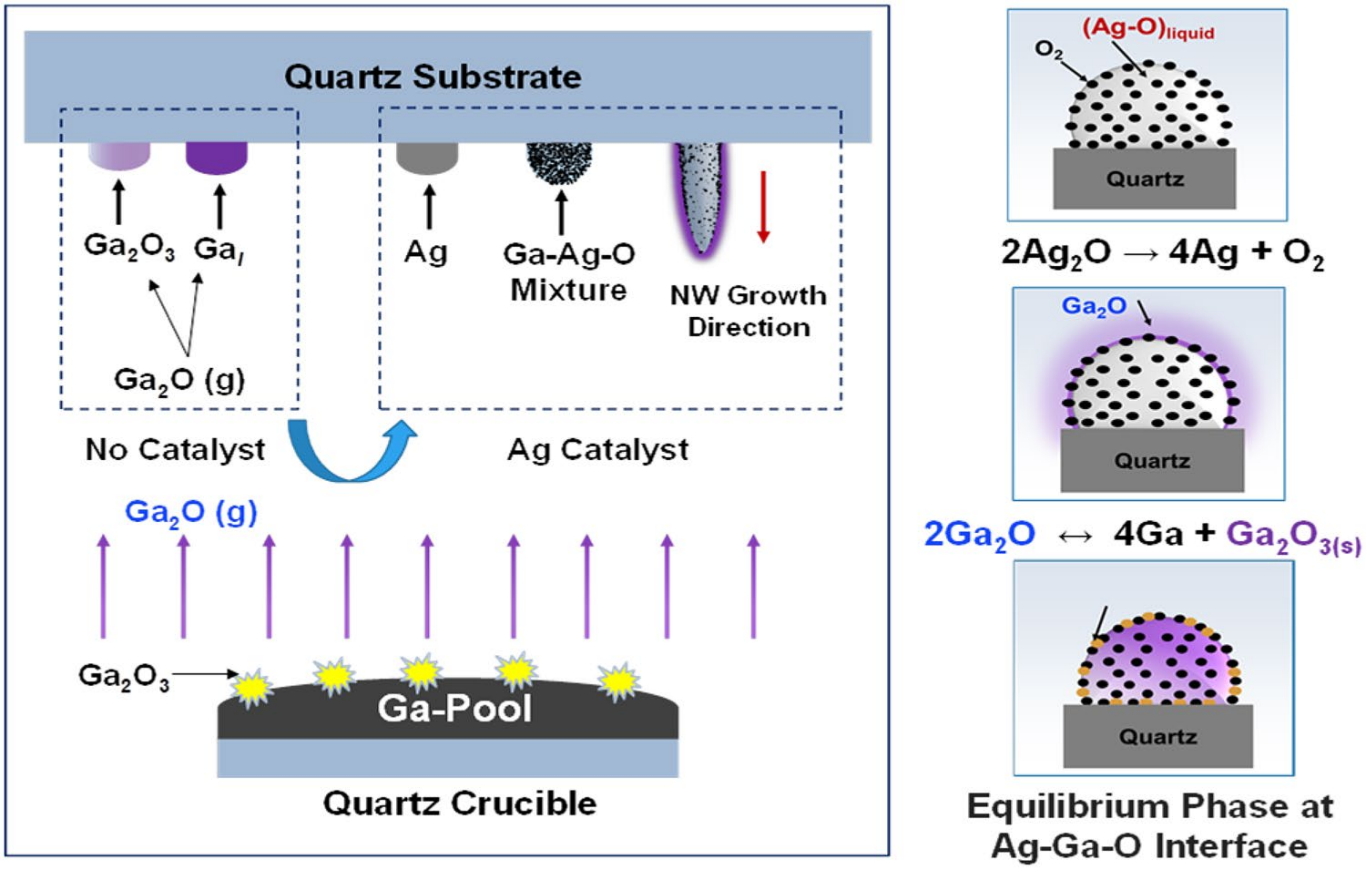
The growth mechanism of Ga_2_O_3_ NWs on a quartz substrate coated with 5 nm Ag film (facing downward). A pool of liquid Ga is heated in a quartz crucible that sits under the quartz substrate with ~ 10 mm gap.

Based on the detailed characterization performed on the oxidation of liquid Gallium (Ga) in a quartz crucible, the presence or absence of an Ag thin film on quartz substrate could help explain the growth mechanism. The growth mechanism is summarized in Fig. 16. Ga is oxidized and forms a solid phase of gallium (III) oxide (Ga_2_O_3_) (Eq. 1)^25,26^. Then, this Ga_2_O_3_ is reduced by liquid metallic gallium and forms a gas phase of gallium suboxide (Ga_2_O) (Eq. 2)^27^. The Ga_2_O gas phase gets transported to cooler regions and decomposes to liquid gallium and Ga_2_O_3_^28,29^, leading to a vapor–liquid–solid (VLS) growth mechanism. In the presence of Ag catalyst, more solid phase of Ga_2_O_3_ is formed.

1 $$ {\text{2Ga }} + \, \left( {{3}/{2}} \right){\text{O}}_{{2}} \to {\text{ Ga}}_{{2}} {\text{O}}_{{{3 }({\text{s}})}} $$

2 $$ {\text{Ga}}_{{2}} {\text{O}}_{{{3}({\text{s}})}} + {\text{ 4Ga}}_{{({\text{l}})}} \leftrightarrow {\text{ 3Ga}}_{{2}} {\text{O}}_{{({\text{g}})}} $$

3 $$ {\text{Ga}}_{{2}} {\text{O}}_{{({\text{g}})}} + {\text{ O}}_{{{2}({\text{g}})}} \to {\text{ Ga}}_{{2}} {\text{O}}_{{{3 }({\text{s}})}} $$

The growth kinetics and the mechanism of incorporation of Ag catalyst to enhance denser and longer β-Ga_2_O_3_ nanowires can be explained using several factors that boost the presence of oxygen in the system. The dewetting of Ag thin films play a critical role in increasing the density of Ag NPs. As the film starts dewetting, the density of Ag NPs increases due to thermal and self-diffusion effects. At higher temperatures above 200 °C , silver oxide decomposes into solid phase of Ag and oxygen molecules (O_2_), which will be partially dissolved in Ag^30^. In addition, the solubility of O_2_ in solid Ag^31^ is much higher than the solubility of oxygen in liquid Ga^26^. Moreover, O_2_ solubility in Ag is significantly increased by increasing the temperature at fixed pressure. For instance, the solubility of O_2_ in Ag is 38.1 ppm at 800 °C and 3000 ppm at 1000 °C at 1 bar of O_2_ pressure^32^. Consequently, oxygen from the furnace atmosphere is dissolved in Ag film. Consequently, there is an availability of oxygen that enhances the growth of Ga_2_O_3_. Furthermore, the enthalpy of formation of the oxidation of Ga is much more negative than that of the oxidation of Ag, at all temperatures. This shows that the spontaneous nucleation of Ga_2_O_3_ begins to occur on Ag-Ga-O liquid mixture, where Ga strips the dissolved O_2_ away from Ag islands that form after dewetting.

On the Ag coated quartz, the vapor phase of gallium suboxide (Ga_2_O) spontaneously separates into Ga_2_O_3_ and liquid Ga (Eq. 2) or reacts with O_2_ dissolved in Ag to form Ga_2_O_3_ (Eq. 1). The Ga_2_O vapor coats the mixture of Ag and Ga_2_O_3_ spontaneously to enhance more Ga_2_O_3_ formation at the surface of Ag. These Ga_2_O_3_ molecules could dissolve in Ag and move to the interface of Ag and Ga_2_O_3_, leading to an equilibrium phase of AgGaO_x_ (Ag–Ga–O phase diagram No. 209084)^33^. At 800 °C, oxygen’s solubility in Ag is highly increased and it contributes to enhanced Ga_2_O_3_ nucleation. However, at higher temperature (1000 °C), the growth saturates and gallium (III) oxide (Ga_2_O_3_) nanowires are enhanced due to higher O_2_ solubility. Therefore, a continuous supply of Ga_2_O and O_2_ in the presence of Ag would increase the growth of Ga_2_O_3_.

### Ag NPs enhances the photosensitivity of Ga_2_O_3_ nanowires

#### Photo-sensitivity at the interface of Ag-free NPs and Ga_2_O_3_ nanowires

The experimental results of the two types of fabricated devices (Ag-free Ga_2_O_3_ on quartz and Ga_2_O_3_ on quartz in the presence of Ag). Generally, the trap states of oxygen generated at the surface of gallium oxide have a large impact on device performance^23^. In Ag-free Ga_2_O_3_ on quartz, the results show weaker response compare to that of Ga_2_O_3_ on quartz in the presence of Ag. Under illumination, the Ag-free Ga_2_O_3_ nanowires had a smaller surface depletion layer due to the insufficient volume of ionized oxygen; hence, most of the photogenerated electron–hole pairs could recombine at the Ga_2_O_3_ nanowire crystal defects, resulting low response photocurrent.

#### Photo-sensitivity of Ag NPs at the interface of Ga_2_O_3_ nanowires

**Figure 17:**
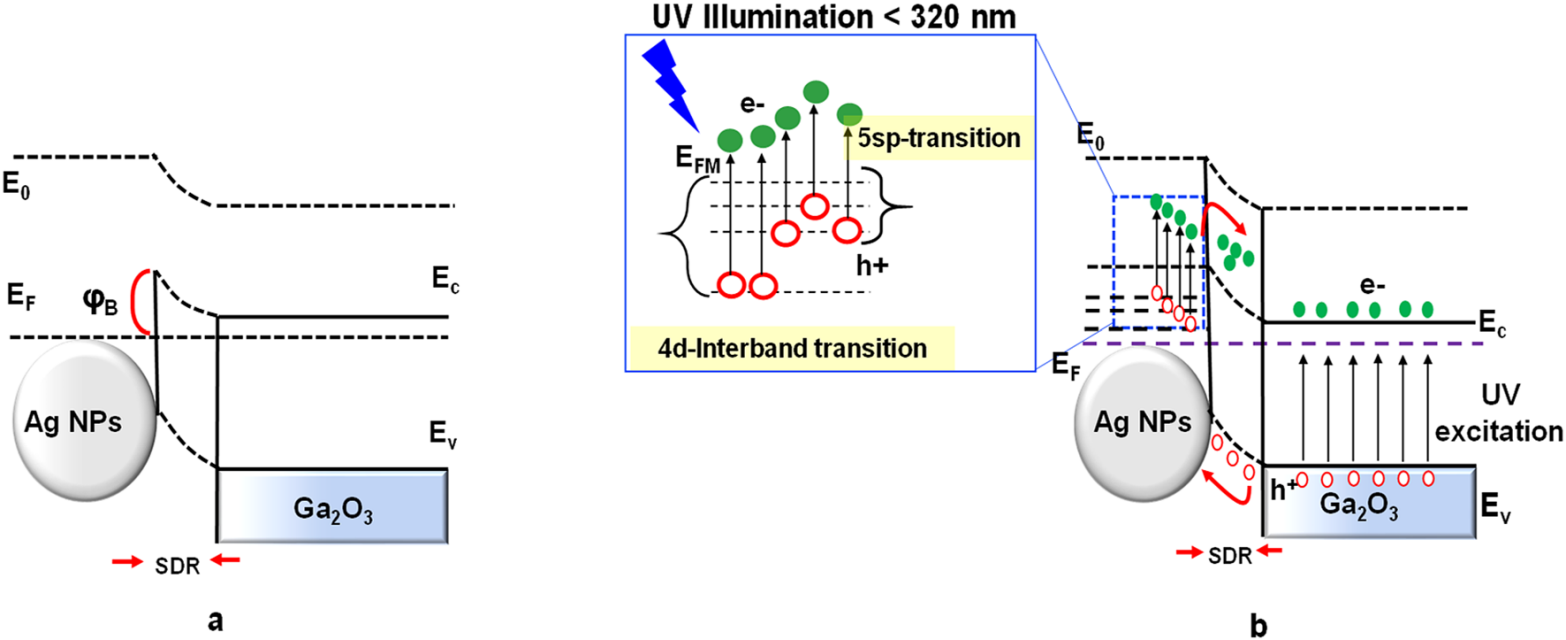
Energy band diagram at the interface of Ag NPs and Ga_2_O_3_ NWs. (**a**) In dark condition, a localized Schottky barrier exists due to the difference in work-function. (**b**) Under UV illumination, the interband transition in Ag NPs enhances the photosensitivity of the UV detection and more photo-generated holes of Ga_2_O_3_ NWs migrate to the surface by band bending.

The experimental results of Ga_2_O_3_ on quartz in the presence of Ag nanoparticles are explicitly explained using schematics of the interface of the materials in the dark and under UV illumination. To illustrate the photodetection mechanism, the energy band diagrams of a β-Ga_2_O_3_ photodetector in the presence of Ag nanoparticles are plotted in Fig. 17.

In β-Ga_2_O_3_ nanowires coated with Ag NPs, the work function (φ_Ga2O3_) and electron affinity (χ_Ga2O3_) of β-Ga_2_O_3_ are 4.11 $\pm$ 0.05 eV and 4.00 $\pm$ 0.05 eV^34^, respectively, which is lower than the work function of Ag (φ_Ag_ = 4.26 eV)^35^. This causes a Schottky barrier (φ_B_ = φ_Ag_ − χ_Ga2O3_ = 0.26 eV) to form. The Schottky barrier prevents the movement of electrons from Ag to Ga_2_O_3_. Consequently, a localized Schottky junction will form a charge depletion region at the interface of Ag NPs and β-Ga_2_O_3_ nanowires. In dark condition, Ag NPs deplete the carriers at the interface. As the width of the depletion layer increases, the depletion region close to Ag NPs increases. Therefore, there is a large depletion width at the interface between Ag NPs and β-Ga_2_O_3_ nanowires. The dark current of the photodetector in β-Ga_2_O_3_ in the presence of Ag NPs thus decreases (Fig. 17a).

Under UV illumination, when the photon energy is larger than the bandgap of Ga_2_O_3_, electron–hole pairs are generated [*hv* → e^-^ + *h*^+^]. Hence electrons move from the valence band to conduction band, leaving behind a hole. Based on traps and photoconduction mechanism, photogenerated carriers instantly increase the carrier density in β-Ga_2_O_3_ nanowires and improve the photocurrent response. Oxygen molecules are absorbed on the surface of Ga_2_O_3_ and are ionized by free electrons [O_2_ + e^-^ → O_2_^-^[ad]]^33,36^. The holes migrate to the surface, accumulate, recombine with adsorbed ionized oxygen and form free oxygen molecules from the surface [O_2_^-^[ad] + h^+^  → O_2_]. The remaining electrons become the majority carriers that contribute to an increase in the photocurrent by generation and recombination until reaching an equilibrium level. If the incident UV light on β-Ga_2_O_3_ in the presence of Ag NPs is below 320 nm, the effect of interband transitions on Ag NPs at the interface of β-Ga_2_O_3_ nanowires allows the transition of highly energetic hot electrons of Ag NPs which excite from the 4d and 5sp bands^20–22^. These hot electrons surmount the small Schottky barrier height and form local band bending downward on Ga_2_O_3_ side to enable the electron transfer from Ag NPs to the conduction band of Ga_2_O_3_ nanowires. Consequently, there is a higher electron density in Ga_2_O_3_ nanowires with Ag NPs compared to Ag-free Ga_2_O_3_ nanowires, leading to a higher photocurrent and hence a more photo-sensitive photodetector (Fig. 17b).

In addition, the efficiency of the device is highly influenced by the presence of oxygen generated at the surface of Ga_2_O_3_ nanowires, causing trap states^23^. These states at the surface of Ga_2_O_3_ nanowires contains highly dense dangling bonds. Therefore, the surface of nanowires has higher sensitivity and better detection mechanism due to the large surface to volume ratio of nanowires and Ag NPs presence. It has been found that the photo-to-dark current ratio of nanowires (2.33 × 10^–7^ A) was higher than the that of thin film (9.16 × 10^–8^ A)^37^.

## Conclusion

In brief, a *β*-Ga_2_O_3_ nanowires for UV photodetector with and without Ag catalyst has been proposed and demonstrated. Silver plays a critical role to enhance the growth mechanism of Ga_2_O_3_ nanowires due to its higher O_2_ solubility and diffusivity. The growth mechanism and characterization of Ga_2_O_3_ nanowires without and with Ag catalyst has been explained. It was found by SAED that the orientation of the nanowires is not affected by the use of Ag catalyst during growth. The ratio of photo-to-dark current without and with Ag catalyst of 4.55 and 38.3, respectively, was achieved, leading to more sensitive detection of UV light. The responsivity curve shows that Ga_2_O_3_ nanowires in the presence of Ag has a cut-off at 320 nm due to the existence of mid-gap state. Hence, the simplicity of this fabrication method suggests a promising device for sensing UV light, particularly for mass production. Our results could offer a promising technique to grow nanowires with high sensitivity and spectral selectivity to UV light.
